# The modulatory role of short-chain fatty acids on peripheral circadian gene expression: a systematic review

**DOI:** 10.3389/fphys.2025.1595057

**Published:** 2025-07-14

**Authors:** Adriano dos Santos, Andrii Vasylyshyn

**Affiliations:** ^1^ Department of Neurology, University of Bërn, Bërn, Switzerland; ^2^ Faculty of Medicine, Ivano-Frankivsk National Medical University, Ivano-Frankivsk, Ukraine

**Keywords:** short-chain fatty acid, SCFA, circadian rhythms, peripheral circadian clock, BMAL1, PER1, PER2, CRY1

## Abstract

Circadian rhythm disruption significantly impacts health and causes a wide range of metabolic, cardiovascular, and psychiatric disorders. Changes in peripheral circadian clock expression are associated with the gut microbiome, particularly via the activity of short-chain fatty acids (SCFAs). The objective of this review is to explore the association between SCFA levels and peripheral circadian gene expression. This review was performed according to PRISMA guidelines. In total, eight studies were included after the PubMed database search and screening process based on inclusion and exclusion criteria. Risk of bias assessment was conducted using the SYRCLE and RoB 2 tools for animal and human studies, respectively. The results showed that propionate, acetate, and butyrate levels correlated with the expression of *PER1*, *PER2*, *BMAL1*, *CRY1*, and *CRY2* in peripheral tissues, including the submandibular gland, liver, kidney, and blood serum. These findings suggest that SCFA supplementation may offer therapeutic potential for individuals with circadian misalignment, such as shift workers or patients with metabolic disorders. Although there was methodological variability among the included studies, it did not significantly compromise the overall quality of the review. The limited availability of human studies (n = 1) represents a significant constraint. Nevertheless, the findings support that SCFA supplementation may serve as a potential strategy for peripheral clock modulation. However, further human trials are needed to validate these findings in clinical settings.

**Systematic Review Registration:** https://osf.io/6kd5q.

## 1 Introduction

Circadian rhythms are inner clocks that are responsible for establishing approximately 24-h cycles and play a vital role in the modulation of various physiological processes in the human body, including sleep, hormone release, and metabolism (Vasey et al., 2021). The central clock is located in the suprachiasmatic nucleus (SCN), a part of the hypothalamus, which receives input from specific cells in the retina called intrinsically photosensitive retinal ganglion cells (ipRGCs) through the retinohypothalamic tract (RHT) (Hastings et al., 2018). This input is subsequently used to structure the light/dark (LD) cycle for the inner body via circadian rhythms (Li and Androulakis, 2021). The presence of circadian genes, including *CLOCK*, *BMAL1*, *PER1*, and *CRY1*, has been reported in many peripheral tissues such as the liver, intestinal epithelium, and pancreas (Richards and Gumz, 2012). Rhythmic expression of these circadian transcriptional factors serves an important role in various cells, where they may act as activators or inhibitors of target genes (Takahashi, 2016). Furthermore, their activity is tissue-dependent; for instance, in hepatocytes, they have been shown to regulate glucose and cholesterol metabolism (Frazier et al., 2023). Although some studies suggest the presence of autonomous clock cells in the liver, the primary regulatory influence appears to stem from clock proteins in extrahepatic tissues (Koronowski et al., 2019). The levels of ghrelin and leptin, commonly referred to as hormones of hunger and satiety, are highly influenced by the peripheral clock (Bodosi et al., 2004). One study has reported reciprocal communication between the central and peripheral clocks (Astiz et al., 2019).

While reciprocal communication between central and peripheral clocks regulates key physiological processes, individual differences in circadian alignment further shape these interactions. For instance, people with a genetic predisposition to function better at night (assessed by a polygenic score) suffer less health damage from night shifts (Akimova et al., 2023). This is largely attributed to chronotype, a genetic tendency for preference in activity and sleep timing. This resilience to disruptions in some individuals stems from a more favorable association between circadian rhythms and nocturnal activity, which minimizes circadian misalignment, a major factor contributing to metabolic and immune dysfunction.

SCFAs, including acetate, propionate, and butyrate, are metabolites produced by gut microbiota through the fermentation of dietary fibers (Blaak et al., 2020). Recent research suggests that SCFAs also influence circadian genes, potentially through their effects on microbiota (Parkar et al., 2019). However, their impact on central clock gene expression appears to be less pronounced compared to their effects on peripheral clocks (Leone et al., 2015).

The present review focuses on the systematic consolidation of evidence across human, animal, and *in vitro* studies on the link between SCFA levels and circadian gene expression in peripheral tissues. Despite growing evidence on the role of SCFAs in circadian regulation, there is a lack of systematic reviews consolidating findings across human, animal, and *in vitro* studies. This review aims to fill this gap by systematically analyzing the evidence on how SCFAs modulate peripheral circadian gene expression. The collected data can reveal molecular pathways crucial for circadian homeostasis and provide a foundation for future studies in this direction.

### 1.1 Environmental and lifestyle contributors to circadian health

In westernized societies, the circadian rhythm disturbances are a common phenomenon caused by lifestyle factors including exposure to artificial light during the evening and night, meal timing, and working conditions (Meléndez-Fernández et al., 2023). Additionally, air quality and environmental noise have been shown to directly affect biorhythms (Fontana et al., 2019; Palanivel et al., 2020), with poor air quality (PM, 2.5) being associated with epigenetic alterations in circadian genes, phase shifts, and decreased rhythmicity in their expression (Palanivel et al., 2020).

Studies have shown that temperature variations can affect circadian-regulated thermoregulation and melatonin secretion, where the former blunts the nocturnal core body temperature reduction and the latter (in addition to bright light) exacerbates the suppression effect (te Kulve et al., 2017; Ishibashi et al., 2010). With respect to noise exposure, high amplitudes and prolonged exposure can change the expression of central circadian genes such as *PER1*, *PER2*, *BMAL1*, and *NR1D1* in the cochlea and inferior colliculus. This disrupts the patterning of circadian regulation in the local environment of these tissues (Fontana et al., 2019).

Notably, while several studies have concluded that exposure to blue light at night from mobile phones drastically disrupts the circadian rhythm by dampening melatonin production, this has been a contested assumption. A recent study conducted by (Bauducco et al., 2024) highlighted a misunderstanding about this phenomenon. The review provides evidence of the overstated hypothesis about the effect of blue light on melatonin and highlights that the correlation between smartphone light exposure at night and sleep quality is weak (Bauducco et al., 2024). Instead, they emphasize that factors such as content engagement and individual susceptibility (e.g., genetic predisposition) may play a more significant role in sleep disturbances.

In contrast, physical activity levels are positively correlated with circadian health. A review of age-related circadian changes highlighted evidence of the resynchronizing effect of exercise on the peripheral circadian clock and the importance of regular physical activity in preventing muscle loss (De Alencar Silva et al., 2021). Morning exercise is associated with better sleep than evening training in research conducted on university students (Chahine et al., 2022).

### 1.2 Chronic circadian misalignment and health risks

Disruptions to circadian rhythm as a result of prolonged light exposure have been associated with many diseases such as sleep disorders, mood disturbances, metabolic dysfunction, and neurodegenerative diseases. Internal clock misalignment, due to factors including shift work, jet lag, and exposure to artificial light, can worsen various conditions such as obesity, diabetes, cardiovascular diseases, and cognitive decline. In addition, this association is bidirectional, with numerous diseases disrupting circadian regulation (Fishbein et al., 2021). Recent studies suggest that night shift workers and individuals with disrupted sleep schedules, including nurses and military personnel, are more susceptible to suffer chronic diseases, such as cardiovascular conditions and cancer (Streng et al., 2022; Samhat et al., 2020). Similarly, chronic jet lag, which often occurs in people traveling across different time zones, significantly affects the inner clock and leads to immune weakening (Khan et al., 2021).

### 1.3 Potential therapies

Although there is currently no universal or standardized treatment for individuals with circadian rhythm misalignment, some therapies have shown positive outcomes across studies, such as afternoon melatonin intake and intermittent bright light exposure (Revell et al., 2006; Dodson and Zee, 2010). Nevertheless, additional interventions are needed, particularly those that may also target endogenous metabolic pathways. A growing body of research has pointed towards the role of the microbiome and nutrition in regulating peripheral clocks.

Circadian rhythms are significantly influenced by genetic and environmental cues, with nutrition having a high impact on clock gene expression and metabolic synchronization across tissues (Rijo-Ferreira and Takahashi, 2019; Pickel and Sung, 2020; Martínez-García et al., 2022). Prebiotics, primarily polysaccharides, enhance the development of beneficial intestinal bacteria through fermentation in the gut microbiota while being indigestible by the human body, whereas probiotics are living microorganisms that are essential for the inhibition of pathogenic bacteria in the intestine (You et al., 2022). The growth of probiotics is facilitated by the presence of prebiotics (You et al., 2022). Postbiotics, active substances, result from probiotic growth (Ji et al., 2023). Probiotics, prebiotics, and postbiotics are associated with modulation of the peripheral clock through the gut microbiota (Tian et al., 2022; Lopez-Santamarina et al., 2023). This outcome can be explained by the gut bacteria that have been reported to be linked to clock genes (Martínez-García et al., 2022). Some studies on the implications of several bacterial families, including Prevotellaceae and *Faecalibacterium*, found that the regulation of hepatic clock genes and gut-liver axis homeostasis is associated with changes in the microbiome (Zeb et al., 2020; Graniczkowska et al., 2023; Zhen et al., 2023).

Microbiota-derived metabolites, such as SCFAs, are formed as a result of dietary fiber fermentation (Besten et al., 2013), which modulate peripheral clocks (Tahara et al., 2018). Similarly, dairy products, particularly butter, contain SCFAs such as butyrate (Danudol and Judprasong, 2022). However, an important consideration is the industrial processing of dairy. Milk fat globule size, which is dependent on homogenization, changes the interaction with the immune system and may affect the activated response via macrophage activation (Michalski, 2007). Moreover, dietary cholesterol (DC) is not intrinsically harmful, but oxidation of dietary cholesterol to oxidized low-density lipoprotein (oxLDL) is a major contributor to cardiovascular disease (CVD) risk (Hong et al., 2023). Lifestyle factors are involved in the conversion between LDL and oxLDL, such as circadian misalignment, sleep deprivation, and dietary oxidative stress (Maiolino et al., 2013). Therefore, the impact of dairy products on circadian and metabolic health is multifaceted, depending not only on their SCFA content but also on the methods of industrial processing and the individual’s broader lifestyle factors.

Acetic acid, propionic acid, isobutyric acid and butyric acid play essential roles in inducing mucus secretion and protective mucosal inflammation (Silva et al., 2020). SCFA levels also fluctuate on a daily basis and this is associated with variations in clock gene expression (Wolever et al., 1997). We hypothesize that SCFAs modulate diurnal rhythms by manipulating the expression of peripheral clock genes, providing promising therapeutic methods. This review explores whether SCFA administration could mitigate circadian rhythm disruptions in shift workers. It examines studies that simulate conditions faced by night shift workers, assessing the effects of SCFAs on circadian modulation and related outcomes.

### 1.4 Mechanisms underlying SCFAs regulation of circadian rhythms

SCFAs play a role in molecular processes that indirectly influence clock genes. The activation of G-Protein-coupled receptors (GPCR), free fatty acid receptor 3 (FFAR3) and free fatty acid receptor 2 (FFAR2), in intestinal and renal epithelial cells triggers intracellular signaling via G proteins (Gi/Go and Gq/11), altering cyclic adenosine monophosphate (cAMP) and calcium levels (Macia et al., 2015). These secondary messengers may modulate the activity of transcription factors such as cAMP-response element binding protein (CREB), which binds to cyclic AMP response element (CRE) in the promoters of *PER1* and *PER2* genes (Travnickova-Bendova et al., 2002).

Circadian rhythms are also regulated through epigenetic modulation, in which SCFAs are involved. Specifically, butyrate and propionate act as potent inhibitors of histone deacetylases (HDACs), which lead to increased acetylation of histones at the H3K18ac and H4K12ac positions (Nshanian et al., 2025). The expression of *BMAL1*, *PER1*, and *CRY1* core clock genes has been observed in intestinal epithelial cells and peripheral tissues, highlighting the capacity of SCFAs to modify chromatin accessibility and reinforce rhythmic gene expression patterns (Firoozi et al., 2024).

FFAR3 and FFAR2 receptors mediate some physiological effects of SCFAs. However, HDAC inhibition appears to occur independently of these receptors. Studies have shown that SCFAs can inhibit HDAC activity in various cell types without requiring FFAR3 or FFAR2 activation (Kobayashi et al., 2018; Andrade-Oliveira et al., 2015).

SCFAs also contribute to the resetting of peripheral clocks through pH-dependent mechanisms. Fluctuations in colonic pH, driven by SCFA production, have been shown to modulate the phase of circadian oscillators, primarily via the TGF-β signaling pathway (Firoozi et al., 2024). The TGF-β signaling pathway is another critical mediator of the effects of SCFAs on circadian gene expression. SCFAs can enhance TGF-β signaling through various mechanisms, including the modulation of receptor availability and intracellular signaling cascades. For instance, insulin signaling has been shown to enhance the delivery of TGF-β receptors to the cell surface, thereby increasing TGF-β responsiveness (Budi et al., 2015). This integration of insulin and TGF-β signaling pathways suggests a complex regulatory network where SCFAs may influence metabolic responses through TGF-β-mediated pathways. Additionally, TGF-β signaling is known to regulate various cellular processes, including cell growth, differentiation, and apoptosis, which are essential for maintaining circadian rhythms (Ikushima and Miyazono, 2011).

**FIGURE 1:**
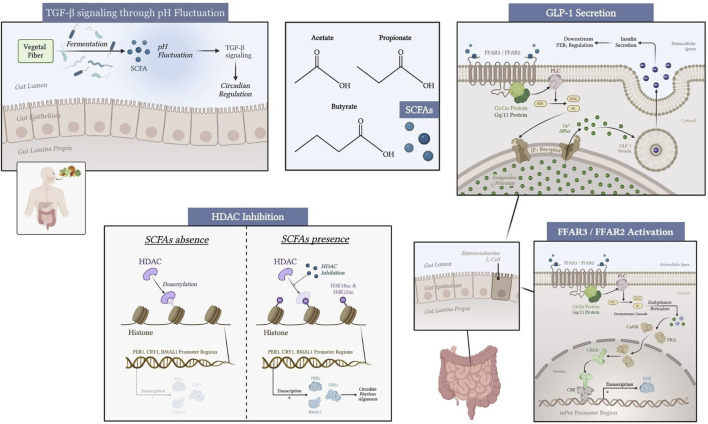
Mechanisms of SCFA modulation of peripheral circadian genes through gut microbiota, FFAR3/FFAR2 receptors, HDAC inhibition, TGF-β signaling through pH fluctuation, and hormone secretion (GLP-1, insulin) in the liver, kidney, and submandibular gland. Created with biorender.com.

SCFA levels exhibit the closest synchronization with circadian rhythms during the host’s active phase (Hibberd et al., 2023). These metabolites interact with FFAR3/2 receptors that are expressed on intestinal L-cells. This initiates a complex intracellular signaling cascade that culminates in the secretion of Glucagon-like peptide-1 (GLP-1) and Peptide YY (PYY). These incretin hormones trigger the release of insulin, a pivotal regulator of systemic metabolic homeostasis. The secretion of GLP-1 from intestinal L-cells is stimulated by SCFAs, particularly butyrate and propionate, leading to enhanced insulin secretion from pancreatic β-cells (Nagata et al., 2024; Budi et al., 2015). Insulin, in turn, modulates *PER2* gene expression to entrain the hepatic clock to align circadian metabolic rhythms with feeding cycles (Tahara et al., 2018). This hormonal interplay is vital for maintaining glucose homeostasis and can influence circadian rhythms by modulating energy metabolism and feeding behavior. Furthermore, the interaction between SCFAs and insulin signaling pathways underscores the importance of gut microbiota in regulating metabolic health and circadian biology (Figure 1).

### 1.5 Beneficial effects of SCFA supplementation

Aside from their role in circadian rhythm modulation, SCFAs can regulate inflammation, the immune system, and metabolic processes. They are implicated in diverse mechanisms that help maintain physiological homeostasis. SCFAs modulate inflammation by inhibiting pro-inflammatory cytokines such as interleukin-6 (IL-6) and tumor necrosis factor alpha (TNF-α) (Olsson et al., 2021). On the other hand, SCFAs promote the expression of anti-inflammatory mediators, including IL-10 and transforming growth factor (TGF-β) (Wang et al., 2017; Vieira et al., 2017). Additionally, SCFAs regulate NF-κB activity, a transcription factor involved in the inflammatory response and responsible for inducing caspase-dependent apoptosis in neutrophils, resolving chronic inflammation (Vieira et al., 2017).

SCFAs activate FFAR3 and FFAR2 (Yang et al., 2020). They participate in immune cell signaling support regulatory T cell (Treg) differentiation and are correlated with immune homeostasis and the regulation of dendritic cell activation, thus preventing excessive immune responses. In addition, acetate stimulates the secretion of IL-22, which plays a role in the host defense and maintenance of fortification of the intestinal barrier (Ouyang and O'Garra, 2019). Accordingly, these different avenues highlight the potential for SCFAs to be used therapeutically in inflammatory and autoimmune diseases.

SCFAs are involved in metabolic health by modulating energy homeostasis and lipid metabolism. In a study on pigs, SCFAs regulated appetite by stimulating the secretion of gut-derived hormones, such as GLP-1 and PYY, which increase satiety (Jiao et al., 2020). Additionally, SCFAs modulate lipogenesis and fat oxidation by upregulating peroxisome proliferator-activated receptor α (PPARα) gene expression, thereby decreasing hepatic fat synthesis and enhancing lipid metabolism (Higashimura et al., 2015). Moreover, SCFAs promote browning of white adipose tissue (WAT), which boosts thermogenesis and energy expenditure (Sahuri-Arisoylu et al., 2016). Hence, SCFAs could serve as potential interventions for obesity and metabolic disorders (Xiong et al., 2022).

In humans, 12-week supplementation of sodium butyrate oral capsules (600 mg/day) has been shown to affect the expression of blood clock genes (*CRY1*, *CRY2*, *PER1*, *BMAL1*) compared to placebo, improve sleep quality, and reduce inflammation, indicating restored peripheral clock function (Firoozi et al., 2024). Similarly, in mice, intraperitoneal administration of butyrate (1,000 mg/kg) for 5 days modulated peripheral circadian gene expression in the liver, further supporting the role of butyrate in regulating systemic circadian rhythms (Leone et al., 2015).

### 1.6 Potential side effects of SCFA supplementation

Although SCFA supplementation is generally considered safe, there is some emerging evidence of potential risks. In preclinical studies, elevated levels of SCFAs have been associated with renal hydronephrosis and hyperplasia in kidney and ureter tissues (Park et al., 2016). Additionally, in *ex vivo* models of ulcerative colitis (UC), SCFAs have been shown to enhance the effector function of activated CD4^+^ T cells, causing acute inflammation and increased risk of liver injury (Gao et al., 2024).

In addition, propionate can disrupt brain neurochemistry by increasing levels of IFN-γ and caspase-3 while reducing levels of noradrenaline, dopamine, and serotonin (Al-Salem et al., 2016). Propionate also elevates glutamate levels and the glutamate/glutamine ratio, as it decreases gamma-aminobutyric acid (GABA), glutamine, and the GABA/glutamate ratio (El-Ansary et al., 2017). These changes have been correlated with neurochemical imbalances related to autism (El-Ansary et al., 2017). Lastly, whereas SCFAs can be beneficial in improving symptoms of UC, they can also trigger gut barrier disruption at the acute inflammatory stage (Trapecar et al., 2020). Whereas SCFAs generated from inulin fermentation demonstrated a potential to prevent type 1 diabetes, direct administration of oral SCFAs had no considerable effect on insulin and blood glucose levels (meta-analysis, 44 RCTs) (Cherta-Murillo et al., 2022). To provide safe and efficacious clinical application, future studies should focus on establishing ideal dosages, long-term metabolic effects, and interactions with pre-existing metabolic conditions. These findings should also be substantiated by high-quality epidemiological research.

## 2 Materials and methods

This systematic review adhered to the guidelines of the Preferred Reporting Items for Systematic Reviews and Meta-Analyses (PRISMA) (Page et al., 2021). The review has been registered in the Open Science Framework (OSF) (Dos Santos and Vasylyshyn, 2025).

### 2.1 Search strategy

Eligible articles were determined through a PubMed database search conducted from December 2024 through January 2025. The search was initially restricted to studies specifically focused on liver circadian gene expression. Due to the lack of adequately designed studies meeting the inclusion criteria, we expanded the search to include studies on peripheral circadian gene expression irrespective of tissue type. The search was conducted using the following keywords: (“Postbiotic” OR “Short Chain Fatty Acids” OR “SCFAs” OR “SCFA”) AND (“gene expression” OR “circadian genes” OR “circadian gene” OR “liver circadian genes” OR “clock genes” OR “Bmal1” OR “Per1” OR “Cry1” OR “Cry2” OR “liver transcriptome” OR “liver circadian clock”). Moreover, manual internet and reference list searches of included studies were performed to identify relevant studies that might have been missed in the keyword search. However, the manual search did not identify any additional articles. The screening of articles was performd using a two-step strategy, in which two independent reviewers screened the approach searched results (abstract and title) for their relevance, as well as full texts of potentially relevant articles using Rayyan software. We resolved any discrepancies in selection via discussion.

### 2.2 Inclusion and exclusion criteria

All articles were published in English over the last 10 years (2015–2025), and the search was limited to free full-text articles. The exclusion criteria consisted of systematic reviews or other secondary literature, experiments with debatable or unreliable methods, and research with insufficient evidence. The inclusion and exclusion criteria applied in this review were designed to isolate studies directly assessing the link between SCFAs and peripheral circadian gene expression. Studies were excluded if they focused exclusively on central circadian regulation, such as SCFA effects on the SCN. While the SCN is the central pacemaker, this review targeted studies exploring peripheral clocks, given the emerging importance of gut-liver-brain signaling in circadian health. Research that examined circadian gene expression without manipulating or evaluating SCFA levels was not included, as it failed to provide conclusive evidence of SCFA’s impact on circadian rhythms.

### 2.3 Data and outcomes extraction

Data extraction was performed independently by two reviewers, recording the following information: author names, year of publication, journal, study title, design, sample characteristics, intervention, instruments of circadian gene expression analysis, and main findings. All discrepancies were resolved through discussion or third-party arbitration.

### 2.4 Risk of bias

The RCT study risk of bias was evaluated by the revised Cochrane risk-of-bias tool for randomized trials (RoB 2). The animal studies were separately evaluated according to the Systematic Review Centre for Laboratory Animal Experimentation (SYRCLE) guidelines. Two authors performed independent evaluation, and any discrepancies were resolved through discussion, with a third reviewer consulted if necessary.

## 3 Results

### 3.1 Study selection

**FIGURE 2:**
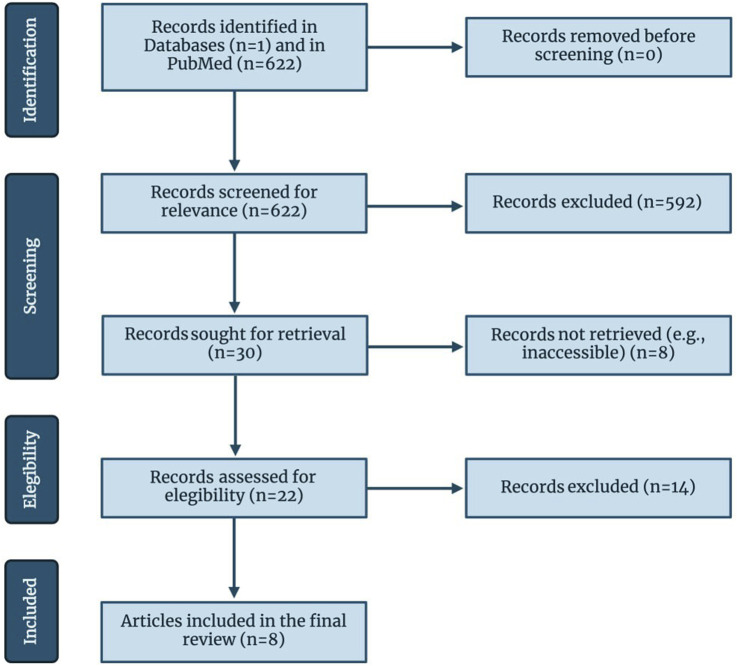
Flow of information through the different phases of the review, following PRISMA guidelines. Created with biorender.com.

A database search yielded 622 studies. After abstract and title screening, 592 articles were excluded, and 30 were retrieved. As a result of retrieval, eight studies were excluded and 22 studies remained for full-text screening. Finally, fourteen studies did not meet the criteria for full-text screening, resulting in a total of eight studies being included in this systematic review. The study selection process is illustrated in Figure 2.

### 3.2 Study characteristics

Eight studies explored the correlation between SCFA concentration and peripheral circadian gene expression levels (Tahara et al., 2018; Firoozi et al., 2024; Ding et al., 2022; Leone et al., 2015; Ryan et al., 2023; Altaha et al., 2022; Fawad et al., 2022; Desmet et al., 2021). Seven experimental animal studies (Tahara et al., 2018; Ding et al., 2022; Leone et al., 2015; Ryan et al., 2023; Altaha et al., 2022; Fawad et al., 2022; Desmet et al., 2021) and one human double-blind randomized controlled trial (Firoozi et al., 2024) were included. Two studies were conducted in Japan (Tahara et al., 2018; Ryan et al., 2023), two in the USA (Leone et al., 2015; Fawad et al., 2022), one in China (Ding et al., 2022), one in Germany (Altaha et al., 2022), one in Belgium (Desmet et al., 2021), and one in Iran (Firoozi et al., 2024). Table 1 summarizes the study characteristics, including authors, year of publication, journal, title, study design, sample, intervention, tools used for circadian gene expression measurement, and key findings.

**TABLE 1:** Study characteristics.

Authors	Year	Journal	Title	Study design	Sample	Intervention	Tools used for circadian genes expression measurement	Key findings
Leone et al. (2015)	2015	Cell Host & Microbe	Effects of Diurnal Variation of Gut Microbes and High-Fat Feeding on Host Circadian Clock Function and Metabolism	Controlled experiment	Gender-matched C57BL/6 mice were divided into 3 groups with 2–3 for microarray analysis. Three groups were germ-free (GF), conventionalized (TF, fecal transplant), and specific pathogen-free (SPF). Male 8–10 weeks-old GF C57BL/6 mice were split into 3 treatment groups, each group consisting of 4 mice	A group of mice received a 37.5% saturated milk fat diet, and the control group received normal rodent chow. 100 μL of saline or butyrate (1,000 mg/kg) in injection was received by 3 treatment groups for 5 days	16S rRNA amplicon sequencing analysis	A high-fat diet has a negative impact on the circadian clock via microbe-dependent metabolites that are affecting it. Loss of SCFAs has been shown to affect circadian liver gene expression and lead to diet-induced obesity
Tahara et al. (2018)	2018	Scientific Reports	Gut Microbiota-Derived Short Chain Fatty Acids Induce Circadian Clock Entrainment in Mouse Peripheral Tissue	Controlled experiment	The sample consisted of 162 mice that included male ICR, C57BL/6J, and male ICR background heterozygous PER2:LUC knock-in	As an antibiotic treatment, metronidazole (1 g/L), ampicillin sodium (1 g/L), neomycin sulphate (1 g/L), and vancomycin hydrochloride (0.5 g/L) were added to the drinking water. Sodium acetate trihydrate, sodium butyrate, sodium propionate, and sodium L-lactate solution (70%) were used as SCFA treatment that has been mixed into the water	Real time polymerase chain reaction (RT-PCR); *In vivo* recording of bioluminescence oscillations in peripheral tissues; *In vitro* recording of the rhythmic expression of Per2	The supplementation of the SCFA has been shown to phase-advance the circadian rhythms in peripheral tissues *in vivo*. It suggests possible solutions to the jet lag treatment. Day-night oscillation of caecal SCFA has been fluctuating and can be connected to the peripheral clock rhythmicity
Desmet et al. (2021)	2021	Nutrients	Time-Restricted Feeding in Mice Prevents the Disruption of the Peripheral Circadian Clocks and Its Metabolic Impact during Chronic Jetlag	Controlled experiment	144 12-week-old wild-type C57BL/6J mice were used	Mice were divided into three equal groups: two jet lag groups, one was on a time-restricted feeding diet and the other was not restricted, and the third group was a control group that was fed a nighttime-restricted feeding diet.	qRT-PCR.	A study reported that the peripheral clock is being modulated via corticosterone levels in plasma that are correlated with the dark/light cycle in the central clock. In the jetlagged mice fed *ad libitum*, the rhythm of *BMAL1* mRNA expression was phase delayed by 5 h and 55 min, with a significantly dampened amplitude compared to the control mice fed a time-restricted feeding diet.
Altaha et al. (2022)	2022	Molecular Metabolism	Genetic and environmental circadian disruption induce weight gain through changes in the gut microbiome	Controlled experiment	Male SCN-specific Bmal1 knock-out mice and their control littermates on a genetic C57BL/6J background were picked to form groups with 3–4 mice/time point/genotype to measure gene expression and 12 mice per group/time/light condition. In other experiments, these samples were used: n = 6 mice/time point/genotype; n = 6 mice/time point/genotype; n = 4–5 mice/time point/light condition; n = 4–5 mice/time point/genotype; N = 5–6 mice/time point/light condition	A group of mice received 100 lux light exposure and had a shift for 8 h every 5th day. The control group was kept under a 12-h light and 12-h darkness schedule	Quantitative real-time PCR (qRT-PCR)	Genetic and environmental circadian rhythm disruption causes arrhythmicity in SCFA fermentation, particularly propionic acid and acetic acid. Mice with central clock disruption showed to have different circadian gene expression in peripheral tissues
Fawad et al. (2022)	2022	Gastroenterology	Histone deacetylase inhibition by gut microbe-generated short chain fatty acids entrains intestinal epithelial circadian rhythms	Сontrolled experiment	In the study, PER2:LUCIFERASE (PER2:LUC) mice that were C57Bl/6J were used. Human Bmal1-luc enteroids and colonoids were generated for *ex vivo* experiments, as well as HDAC3-inducible knockout enteroids	Mouse enteroids and human enteroids were exposed to SCFAs acetate, butyrate, propionate and isovalerate	Bioluminescence recording	Circadian genes in the intestinal tissues are being affected directly with SCFA, even without receiving input from the central clock or any other peripheral tissues
Ding et al. (2022)	2022	Frontiers in Nutrition	A high-fat diet disrupts the hepatic and adipose circadian rhythms and modulates the diurnal rhythm of gut microbiota-derived short-chain fatty acids in gestational mice	Сontrolled experiment	80 female C57BL/6 mice in the gestational stage	The high-fat diet group received a diet in which 60% of calories were fat, whereas the control group had 15.8% fat calories in their diet.	Reverse transcription quantitative PCR (RT-qPCR)	The microbiota-derived SCFAs are influenced by a high-fat diet and are associated with liver circadian gene expression changes
Ryan et al. (2023)	2023	Frontiers in Nutrition	Euglena gracilis-derived β-glucan paramylon entrains the peripheral circadian clocks in mice	Сontrolled experiment	6–8 weeks old male ICR mice and 8–12 weeks old male heterozygous PER2:LUC knock-in mice with ICR backgrounds. For real-time RT-PCR analysis, 8 mice were taken. Another group of mice was divided into the control and paramylon groups: the control group consisted of 8, and the paramylon group consisted of 4	Euglena (50 mg)/paramylon (50 mg) to the vehicle (10 mg/kg body weight) was supplemented via oral administration. 1 g/L of metronidazole (Fujifilm Wako Pure Chemical Co., Osaka, Japan), 1 g/L ampicillin sodium (Fujifilm Wako Pure Chemical Co.), 1 g/L neomycin sulfate (Fujifilm Wako Pure Chemical Co.), and 0.5 g/L of vancomycin hydrochloride (Fujifilm Wako Pure Chemical Co.) were used as an Antibiotic treatment and added into water	RT-PCR; *In vivo* bioluminescence imaging	Paramylon can possibly mediate clock gene expression by SCFAs and may have a similar effect as SCFAs direct intake
Firoozi et al. (2024)	2024	Lipids in Health and Disease	Effects of short-chain fatty acid-butyrate supplementation on expression of circadian-clock genes, sleep quality, and inflammation in patients with active ulcerative colitis: a double-blind randomized controlled trial	Randomized controlled trial	16 women and 20 men with mild to moderate active ulcerative colitis	The intervention group with 18 individuals was receiving 600 mg/d sodium butyrate in capsule form for 12 weeks. The control group with 18 individuals received a placebo (600 mg/d of rice starch) for 12 weeks	qRT-PCR.	*CRY1*, *CRY2*, *PER1*, and *BMAL1* gene expression increased after SCFA supplementation. *PER2* and *CLOCK* gene expression did not show a big difference between the intervention and control groups

Different types of real-time PCR (RT-PCR) (Tahara et al., 2018; Firoozi et al., 2024; Ding et al., 2022; Ryan et al., 2023; Altaha et al., 2022; Desmet et al., 2021), bioluminescence imaging (Tahara et al., 2018; Ryan et al., 2023; Fawad et al., 2022), and 16S rRNA amplicon sequencing (Leone et al., 2015) are three distinct methodologies employed to measure circadian gene expression in the included studies.

Bioluminescence imaging using PER2LUC reporter mice offers a real-time, *in vivo* assessment of circadian gene expression. This method exploits the bioluminescent properties of luciferase, allowing researchers to monitor the oscillation of *PER2* expression in living organisms. However, this method may not provide the same level of specificity as qRT-PCR, as it measures the overall bioluminescence rather than specific mRNA levels (Baba et al., 2017).

The 16S rRNA sequencing approach provides a comprehensive view of microbial diversity and can reveal temporal changes in microbial communities in relation to circadian cycles (Milićević et al., 2024). However, it is important to note that while 16S rRNA sequencing can indicate community composition, it may lack the resolution to differentiate closely related species due to the conserved nature of the 16S rRNA gene (Ono et al., 2015). Furthermore, the methodology is primarily focused on microbial analysis and does not directly measure circadian gene expression in host tissues (Taylor et al., 2020).

### 3.3 SCFA measurement methods, SCFA concentrations, and changes in peripheral circadian gene expression

SCFA dosages varied widely across studies, ranging from millimolar concentrations in animal trials (Tahara et al., 2018; Ding et al., 2022; Leone et al., 2015; Ryan et al., 2023; Altaha et al., 2022; Fawad et al., 2022; Desmet et al., 2021) to 600 mg/day in human supplementation studies (Firoozi et al., 2024). These variations in dosage make it challenging to accurately compare study results. The mode of delivery also varied, including: oral supplementation in drinking water (Tahara et al., 2018), direct intraperitoneal injection (Leone et al., 2015), and dietary incorporation through SCFA-enriched diets (Ding et al., 2022).

Circadian rhythms shift in response to external and internal factors, with phase advances occurring when biological cycles start earlier than usual, and phase delays when they are pushed later in time. All animal studies reported delays in the phase of peripheral circadian expression following elevation in SCFA levels (Tahara et al., 2018; Ding et al., 2022; Leone et al., 2015; Ryan et al., 2023; Altaha et al., 2022; Fawad et al., 2022; Desmet et al., 2021). However, two studies have shown phase advancement of circadian genes in the kidney and submandibular gland (Tahara et al., 2018; Ryan et al., 2023). While one study assessed circadian regulation through direct gene expression analysis, the other examined metabolic markers (SCFA levels), which are known to influence downstream gene expression. Both approaches offer complementary insights into the role of SCFAs in circadian rhythm modulation (Tahara et al., 2018). observed a phase advancement of *PER1*, *PER2*, *BMAL1*, *CRY1*, and *NR1D1* in the kidney, as well as some phase advancement of *PER2* and *CRY1* in the submandibular gland following SCFA supplementation.

In contrast (Ryan et al., 2023), reported phase-advanced PER2:LUC activity in the kidney and submandibular gland in mice administered paramylon, a β-glucan derived from *Euglena gracilis* which is known for its effects on gut microbiota and modulation of SCFA production. While butyrate and propionate levels were higher in the paramylon group, total SCFA concentration remained unchanged. The change reflected a shift in composition rather than an overall increase (Ryan et al., 2023). Similarly, a high-fat diet intervention resulted in increased propionic acid, valeric acid, and hexanoic acid levels. These molecules were associated with oscillations in *CLOCK*, *BMAL1*, *PER2*, *CRY2*, and *NR1D1* genes in hepatocytes and WAT (Ding et al., 2022). However, another study found that direct SCFA administration did not induce changes in hepatic circadian gene expression (Tahara et al., 2018), which indicates that additional dietary or metabolic factors may modulate this effect. Intraperitoneal injection of butyrate (1,000 mg/kg) was associated with an increased *PER2*:*BMAL1* mRNA ratio (Leone et al., 2015). Accordingly, SCFAs influence hepatic circadian rhythms through multiple interconnected pathways, including host metabolic state, gut microbiota rhythmicity, and epigenetic regulation.

(Altaha et al., 2022) employed a multiple reaction monitoring method alongside the 3-nitrophenylhydrazine (3-NPH) method to measure fecal SCFA concentration. Their findings indicated no rhythmicity in the total SCFA levels in BMAL1SCNfl/- mice, whereas butyric acid and valeric acid showed rhythmicity compared to the control group. In the *ex vivo* study performed by (Fawad et al., 2022), PER2LUC mouse enteroids showed 2–15 h of delay in phase due to exposure to acetate (10mM, median = 3.72h, *p* < 0.05), butyrate (1mM, median = 13.35h, *p* < 0.05), propionate (1mM, median = 3.26h, *p* < 0.05; 10mM, median = 11.74h, *p* < 0.05), and isovalerate (0.5mM, median = 5.1h, *p* < 0.05; 1mM, median = 8.1h, *p* < 0.05).

Similar to the studies with paramylon intervention (Ryan et al., 2023) and high-fat diet (Ding et al., 2022; Desmet et al., 2021) measured fecal SCFAs using the gas chromatography method. The results demonstrated a delay of 7 h and 4 min in the phase rhythmicity of fecal propionate relative to the control group. However, the same study revealed no statistically significant changes in fecal acetate and butyrate in jet-lagged mice with time-restricted feeding (acetate: ZT 6h39, *p*_cosinor_ < 0.05; butyrate: ZT 3h13, *p*_cosinor_ < 0.001) compared with the control group (acetate: ZT 4h28, *p*_cosinor_ < 0.01; butyrate: ZT 3, *p*_cosinor_ < 0.001). Additionally, the study observed phase advancements in the expression of *BMAL1* mRNA and *NR1D1* mRNA in jet-lagged mice that did not undergo time-restricted feeding. Specifically, *BMAL1* mRNA expression shifted by 5 h and 55 min, whereas *NR1D1* mRNA expression advanced by 6 h and 31 min.

Finally, in the included human randomized controlled trial (RCT) study (Firoozi et al., 2024) on the effect of butyrate supplementation on circadian gene expression, more than a three-fold increase in the *CRY1* expression (*CRY1* fold change: 2.22 ± 1.59 vs 0.63 ± 0.49, *p* < 0.001) was discovered in blood samples after 600 mg/d sodium-butyrate capsule supplementation for a 12-week period. Moreover, more than a two-fold increase was present in *CRY2* (*CRY2* fold change: 2.15 ± 1.26 vs 0.93 ± 0.80, *p* = 0.001), *PER1* (*PER1* fold change: 1.86 ± 1.77 vs 0.65 ± 0.48, *p* = 0.005), and *BMAL1* (*BMAL1* fold change: 1.85 ± 0.97 vs 0.86 ± 0.63, *p* = 0.001) after intervention. Detailed information regarding the SCFA measurement tools, SCFA concentrations, and changes in peripheral gene expression are presented in Table 2.

**TABLE 2:** SCFA measurement methods, SCFA concentrations, and changes in peripheral circadian gene expression.

Study	SCFA measurement method	SCFA concentration	Peripheral circadian gene expression changes
Tahara et al. (2018)	Caecal SCFA was measured by ion-exclusion HPLC	Increased via oral supplementation (2–3 times higher compared to levels in the colon in the control group)	All *PER1*, *PER2*, *BMAL1*, *CRY1*, and *NR1D1* in the kidney and some of the *PER2* and *CRY1* in the submandibular gland were phase advanced after SCFA administration. No reported changes in liver circadian gene expression
Ding et al. (2022)	SCFAs (acetic acid, propionic acid, butyric acid, valerate acid, isobutyric acid, isovaleric acid, hexanoic acid) were extracted from frozen liver tissue analyzed using HP-FFAP capillary column and measured with gas chromatography mass spectrometry (GC-MS)	Propionic acid, valeric acid, and hexanoic acid increases were reported in the interventional group	Rhythmic oscillations, causing phase advance in liver and white adipose tissues, *CLOCK*, *BMAL1*, *PER2*, *CRY2*, and *NR1D1* after feeding high fat were observed
Leone et al. (2015)	Cecal or fecal SCFA was measured using Varian Saturn 2000 GC-MS-MS.	Increase of butyrate levels by intraperitoneal administration (1,000 mg/kg)	Increased *PER2*:*BMAL1* mRNA ratio after intervention compared to the control group
Ryan et al. (2023)	Cecum SCFA levels were measured using gas chromatography	No difference in total SCFA concentration between groups. Butyric and propionic acid levels were higher in the paramylon group	Paramylon group showed significantly phase-advanced PER2:LUC activity in the kidney and submandibular gland
Altaha et al. (2022)	Fecal SCFA concentration was measured with the multiple reaction monitoring method and the 3-NPH method	No rhythmicity of total SCFAs was found in BMAL1SCNfl/- mice. In control and BMAL1SCNfl/- mice, butyric acid and valeric acid showed rhythmicity	In BMAL1SCNfl/- mice, all clock genes were advanced in the jejunum, cecum, and proximal colon (*BMAL1*: 2.7h, Per2: 3.6h, *NR1D1*: 5.7h)
Fawad et al. (2022)	Nuclear magnetic resonance (NMR) metabolic profiles were used to approximately evaluate total SCFA concentration	Increase in SCFAs levels after acetate, butyrate, propionate, and isovalerate exposure to PER2:LUC enteroids	2–15 h of delay in phase in PER2:LUC mouse enteroids
Desmet et al. (2021)	Fecal SCFA concentration was identified using gas chromatography–flame ionization detector	Control group and jet-lagged mice with time-restricted feeding did not show significant changes in fecal acetate and butyrate. In the jet-lagged mice without time-restricted feeding, the fecal propionate concentrations showed a delay in phase rhythmicity of 7 h and 4 min compared to the control group	*BMAL1* mRNA expression in the mucosa of the distal colon was phase delayed by 5 h and 55 min in jet-lagged, not time-restricted, feeding mice compared to the control group. *NR1D1* mRNA expression was phase delayed by 6 h and 31 min in jet-lagged, not time-restricted, feeding mice compared to the control group
Firoozi et al. (2024)	No tools were used	Increased by 600 mg/d sodium-butyrate capsule supplementation for 12 weeks	A significant increase compared to the placebo group in the expression of *CRY1*, *CRY2*, *PER1*, and *BMAL1* was found in serum isolated from blood samples. Not significant changes were reported in *PER2* and *CLOCK* expression

The included studies showed significant variability in the types of samples analyzed for circadian gene expression. While some studies focused exclusively on hepatic tissue (Ding et al., 2022), others examined intestinal tissues (Desmet et al., 2021), and even submandibular glands (Ryan et al., 2023). Tissue-specific circadian clocks have different entrainment mechanisms and SCFA receptor expression profiles (FFAR3/2), which makes direct comparisons more difficult. This variability, however, represents the systemic aspect of circadian control.

### 3.4 Risk of bias

Judgment: L: Low Risk of Bias U: Unclear Risk of Bias H: High Risk of Bias. Domains: D1: Sequence generation D2: Baseline characteristics D3: Allocation concealment D4: Random housing D5: Blinding of caregivers D6: Random outcome assessment D7: Blinding of outcome assessor D8: Incomplete outcome data D9: Selective outcome reporting D10: Other sources of bias.

The animal studies included in the review were subsequently analyzed for a risk of bias across 10 domains, as summarized in Table 3. Two domains, random housing and incomplete outcome data, reported a low risk of bias across the included studies (Tahara et al., 2018; Ding et al., 2022; Leone et al., 2015; Ryan et al., 2023; Altaha et al., 2022; Fawad et al., 2022; Desmet et al., 2021). The only domain that demonstrated a high risk of bias in four cases (Ding et al., 2022; Leone et al., 2015; Altaha et al., 2022; Fawad et al., 2022) was the blinding of outcome assessors. Numerous domains have an unclear risk of bias, primarily because of the lack of detailed methodology regarding randomization processes in sequence generation (Tahara et al., 2018; Leone et al., 2015; Altaha et al., 2022; Fawad et al., 2022) and outcome assessment (Tahara et al., 2018; Ding et al., 2022; Leone et al., 2015; Ryan et al., 2023; Altaha et al., 2022; Fawad et al., 2022). Domain 5, which assessed the blinding of personnel and researchers, was particularly problematic, as none of the included studies explicitly stated that animal caretakers were blinded to the treatment groups. As a result, an unclear or high risk was reported in all included animal studies (Tahara et al., 2018; Ding et al., 2022; Leone et al., 2015; Ryan et al., 2023; Altaha et al., 2022; Fawad et al., 2022; Desmet et al., 2021). In circadian studies where animals may be subjected to feeding manipulations or altered light schedules, a lack of blinding could lead to subtle differences in care or handling. Importantly (Leone et al., 2015), conducted a study using intraperitoneal SCFA injections, which may have increased the likelihood of unblinded handling, as researchers administering the injections would inevitably know the treatment group. There were similar issues in domain 7, which corresponds to blinding of the outcome assessment. Most animal studies used RT-PCR (Tahara et al., 2018; Ding et al., 2022; Ryan et al., 2023; Altaha et al., 2022; Desmet et al., 2021) or bioluminescence imaging (Tahara et al., 2018; Ryan et al., 2023; Fawad et al., 2022). These techniques require multiple processing steps, where awareness of group identity can influence data handling or interpretation. Explicit statements describing that laboratory staff analyzing the samples were blinded were not present in all animal studies (Tahara et al., 2018; Ding et al., 2022; Leone et al., 2015; Ryan et al., 2023; Altaha et al., 2022; Fawad et al., 2022; Desmet et al., 2021); therefore, these domains were frequently marked as unclear or high risk of bias. This is especially critical in studies such as (Ryan et al., 2023), where complex multi-tissue sampling and imaging increase the opportunity for confirmation bias if blinding is not maintained. The SYRCLE guidelines do not specify an overall evaluation of the risk of bias; therefore, the present systematic review does not provide an overall risk of bias for animal studies.

**TABLE 3:** SYRCLE risk of bias.

Authors	D1	D2	D3	D4	D5	D6	D7	D8	D9	D10
Tahara et al. (2018)	U	L	U	L	U	U	U	L	L	L
Ding et al. (2022)	L	L	L	L	U	U	H	L	U	L
Leone et al. (2015)	U	L	L	L	U	U	H	L	U	L
Ryan et al. (2023)	L	L	U	L	U	U	U	L	L	L
Altaha et al. (2022)	U	U	L	L	U	U	H	L	U	U
Fawad et al. (2022)	U	U	L	L	U	U	H	L	U	U
Desmet et al. (2021)	L	L	L	L	U	L	U	L	L	L

Domains: D1: Bias arising from the randomization process. D2: Bias due to deviations from intended intervention. D3: Bias due to missing outcome data. D4: Bias in measurement of the outcome. D5: Bias in selection of the reported result. Judgment: H - High Risk, U - Unclear Risk, L - Low Risk.

The human RCT study (Firoozi et al., 2024) was graded as having a low risk of bias after evaluation using the RoB 2 tool (Table 4). However, there was an uncertain risk of missing outcome data due to the sample size (n = 36) and insufficient dropout reporting, which could have an impact on the effect estimates and generalizability.

**TABLE 4:** RoB 2 Risk of bias.

Study	D1	D2	D3	D4	D5	Overall
Firoozi et al. (2024)	L	L	U	L	L	L

## 4 Discussion

This systematic review explores how SCFAs influence peripheral circadian gene expression. The included studies indicate that rhythmicity in SCFA levels is correlated with peripheral circadian gene expression and can be affected by oral or intraperitoneal supplementation with SCFA. These results provide evidence for the modulation of clock genes in the liver, kidney, submandibular gland, blood serum, jejunum, cecum, proximal colon, and WAT after changes in butyrate, acetate, and propionate concentrations. Moreover, all animal studies (Tahara et al., 2018; Ding et al., 2022; Leone et al., 2015; Ryan et al., 2023; Altaha et al., 2022; Fawad et al., 2022; Desmet et al., 2021) show phase advancement in circadian rhythms after an increase in SCFA levels, and a human study (Firoozi et al., 2024) showed a 3-fold increase in *CRY1* expression levels and a 2-fold increase in *CRY2*, *PER1*, and *BMAL1* expression.

### 4.1 Inconsistencies among tissues

The correlations between specific tissues and SCFA were inconsistent, which indicates that SCFA effects on circadian rhythms may be tissue-specific. These effects could be mediated by distinct local signaling pathways, microbiota compositions (e.g., Prevotellaceae and Faecalibacterium), and variations in SCFA receptor expression (Zeb et al., 2020; Graniczkowska et al., 2023). This might be due to several reasons such as the heterogeneous distribution of receptors, SCFA uptake and metabolism, neuronal and hormonal signaling, and local metabolic states.

SCFAs shift circadian phases in the kidney and submandibular gland but not the liver, owing to greater expression of FFAR3/2 in those tissues. Furthermore, SCFAs stimulate GLP-1 secretion and subsequent insulin release, which has a major impact on insulin-sensitive tissues. In contrast, organs with superior innervation, such as kidneys, are more sensitive to the activation of the sympathetic nervous system (Tahara et al., 2018). SCFAs, such as butyrate and propionate, exert their effects primarily through the activation of GPCRs, notably FFAR2 and FFAR3, which are involved in the regulation of metabolic hormones like GLP-1 (Tolhurst et al., 2012; Kasubuchi et al., 2015). While this activation can enhance insulin sensitivity and promote satiety, excessive SCFA levels may lead to dysregulation of these hormones. For example, overactivation of FFAR2 can result in impaired glucose homeostasis, potentially contributing to insulin resistance and metabolic syndrome (Tolhurst et al., 2012; Kopczyńska and Kowalczyk, 2024). Furthermore, the chronic stimulation of these receptors may lead to desensitization, diminishing their effectiveness over time and possibly exacerbating metabolic disorders (Kasubuchi et al., 2015).

The SCFA levels in the gastrointestinal tract of mice are correlated with diurnal rhythms, paralleling feeding times. These rhythms regulate the release of hormones, such as ghrelin, with SCFAs inhibiting their release in the colon at specific times of the day. This effect is independent of the core clock gene *BMAL1*, which indicates that other clock genes may play a role in local entrainment (Segers et al., 2020).

SCFAs mainly regulate circadian clocks in the intestine, with a weaker impact on other organs. Butyrate synchronizes circadian gene expression in intestinal epithelial cells via HDAC3 inhibition, as shown in murine and human organoid models (Ma et al., 2023; Wu et al., 2020). This effect relies on the high local SCFA concentrations in the colon (∼20–75 mM) compared to much lower plasma levels (∼0.1 mM), limiting systemic impact (Rangan and Mondino, 2022). While SCFAs function as HDAC inhibitors, evidence suggests that epigenetic modulation of circadian rhythms varies by tissue. In the intestine, SCFA-driven circadian regulation primarily occurs through HDAC inhibition (Fawad et al., 2022), while in extraintestinal tissues, it appears to be mediated by NAD+/SIRT1 signaling, linking circadian control to cellular energy metabolism rather than direct HDAC inhibition (Etchegaray and Mostoslavsky, 2016). SCFAs are potent inhibitors of HDACs, leading to increased acetylation of histones and subsequent alterations in gene expression. This mechanism is crucial for the differentiation of regulatory T cells and the modulation of inflammatory responses (Tahara et al., 2018). However, excessive HDAC inhibition can disrupt normal cellular functions and lead to aberrant gene expression patterns. For instance, prolonged SCFA supplementation may result in the upregulation of pro-inflammatory genes, which could counteract the intended anti-inflammatory effects of SCFAs (Fawad et al., 2022). Additionally, the dysregulation of circadian genes due to altered histone acetylation could lead to further disruptions in circadian rhythms, compounding the risks associated with SCFA supplementation (Tahara et al., 2018; Jochum et al., 2022).

SCFAs also selectively activate PPARγ in the intestinal epithelium, inducing genes like *ANGPTL4*, a specificity not observed in other tissues, such as adipocytes, suggesting that SCFA act as selective PPARγ modulators (SPPARM) (Alex et al., 2013). In contrast, hepatic rhythms depend mostly on insulin and feeding cycles, with Per1 responding to postprandial insulin, which is independent of SCFAs (Finger et al., 2020). Likewise, SCFAs do not directly affect central clock genes in the brain, which are regulated by light and neuroendocrine signals (Hastings et al., 2018). One study showed that butyrate injection had no significant effect on the *PER2:BMAL1* ratio in the mediobasal hypothalamus, indicating that this brain region may be less responsive to peripheral SCFA signaling (Leone et al., 2015). There is also contrary evidence suggesting that SCFAs or microbiota-derived metabolites influence brain functions, such as anxiety and sleep. For instance (Miyazaki et al., 2014), proved that probiotics like *Lactobacillus* ameliorated circadian rhythms and sleep in mice. This potential indirect effect of SCFAs on the brain’s circadian clock warrants further research (Miyazaki et al., 2014).

Host metabolic state is a key determinant of SCFA action, as insulin resistance and obesity have been demonstrated to affect SCFA responsiveness, perturbing butyrate and propionate circadian oscillations in high-fat diet models. Hepatic circadian regulation is also modulated by bile acid synthesis and the activation of nuclear receptors (e.g., FXR and LXR) by these bile acids. Moreover, the rhythmicity of the gut microbiota follows circadian cycles in SCFA production, which reaches the peak during the active phase in rodents. A study confirmed the implication of microbiota in integrating signals into biorhythms, as germ-free mice (GF) disrupted hepatic clock gene expression (*BMAL1*, *NR1D1*) (Montagner et al., 2016). When SCFA supplementation is misaligned with the body’s natural circadian rhythms—such as in night shift workers or individuals with irregular eating patterns—this could exacerbate circadian misalignment and lead to metabolic dysregulation (Desmet et al., 2021; Ding et al., 2022). For example, the timing of SCFA intake may disrupt the diurnal rhythms of gut microbiota and their metabolites, leading to further complications in metabolic health (Zhen et al., 2022).

SCFAs exert tissue-specific effects on circadian rhythms, primarily limited to intestinal tissues due to their high local concentrations and selective receptor activation. While they may indirectly influence liver metabolism via enteroendocrine or immune pathways, their direct impact on circadian regulation in other organs, like the liver or brain, is not so significant. Thus, further research is required.

### 4.2 Microbiota relevance on circadian rhythms

Bidirectional crosstalk between the gut microbiota and the host circadian clock has been previously reported (Pearson et al., 2020). Night shift work negatively impacts health and leads to circadian rhythm disruption, causing relative abundance in *Faecibacterium* and *F. prausnitzi* when compared to individuals working day shifts (Mortaş et al., 2020). In a recent narrative review, the positive effect of probiotics, such as *Lactobacillus acidophilus* and *Bifidobacterium infantile*, on circadian rhythms in night shifters with dysbiosis was examined by targeting the gut-microbiome-brain axis (Lopez-Santamarina et al., 2023). Therefore, it might be possible to offer therapy to night workers with circadian rhythm disruptions. Cheng et al. investigated the effect of the beta-glucan and inulin supplementation on mice with induced circadian rhythm misalignment and reported the possibility of targeting the peripheral clock using a dietary approach (Cheng et al., 2020). In a review by (Markowiak-Kopeć and Śliżewska, 2020) on the effect of probiotic supplementation on SCFA production in the human microbiome, it was reported that probiotics, including *Lactobacillus paracasei* ssp. *paracasei BCRC 12188, Lactobacillus plantarum BCRC 12251, Streptococcus thermophilus BCRC13869,* and *L. plantarum P-8* (Lp-8), were shown to increase SCFA levels *in vivo* and *in vitro*.

**FIGURE 3:**
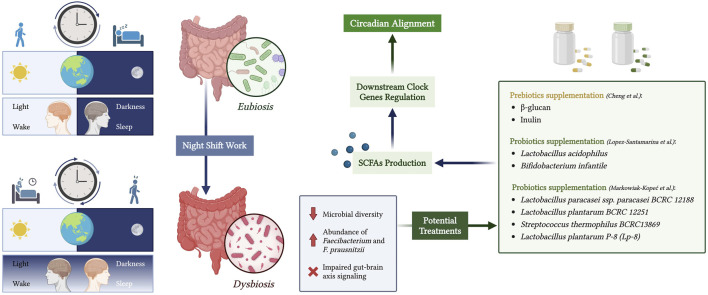
Modulation of peripheral circadian rhythms through the gut microbiota-brain axis in night shift workers. Created with biorender.com.

This data is particularly useful in the context of this review, as it suggests an explanation of how probiotics, by modulating SCFAs, could regulate peripheral circadian gene expression. The molecular pathway of the gut-circadian clock axis could explain further pharmacotherapy or dietary approaches to target circadian rhythm misalignment in nurses, military personnel, and other individuals working inconvenient hours. The potential modulation of peripheral circadian gene expression by probiotic intake is shown in Figure 3.

### 4.3 SCFAs applications and factors to be considered

Another systematic review that included 39 studies explored how resistant starch intake affects SCFA production in healthy individuals and people with medical conditions (Sobh et al., 2021). SCFA production levels increased in 70% of the studies, and most of them reported tolerability in both the healthy and medical condition groups (Sobh et al., 2021). Natural dietary sources of SCFAs may also affect their levels in the body; for instance, a 12-week whole-grain diet intervention was associated with a significant increase in propionate (Vetrani et al., 2015). Similarly, another study that investigated the effects of a high SCFA diet, which included an additional 20 g of high-amylose maize starch and an apple cider vinegar drink instead of a placebo, reported increased plasma concentrations of propionate and butyrate, as well as elevated fecal SCFA levels (Gill et al., 2022). Modulation of peripheral circadian clocks could be particularly useful for metabolic disorders, as it has been previously reported that circadian rhythm disruption is linked to obesity, diabetes, and cardiovascular disorders (Baidoo and Knutson, 2023). Time-restricted feeding, which has been shown to affect jet-lagged mice via the corticosterone modulatory effect on peripheral circadian gene expression (Desmet et al., 2021), was also studied in a recent review by (Roth et al., 2023) and revealed that time-restricted feeding positively impacts metabolic health.

SCFAs have garnered attention for their potential therapeutic benefits, particularly in modulating immune responses and influencing metabolic pathways. However, treatment with SCFAs is not without risks, which can manifest in various physiological and pathological contexts. One significant concern is the potential for SCFA treatment to exacerbate certain neurological conditions. For instance, excessive propionate intake has been linked to an increased risk of Alzheimer’s disease (AD) in healthy individuals, as it may lead to microglial activation and neuroinflammation (Xie et al., 2023; Killingsworth et al., 2021). This suggests that while SCFAs can have beneficial effects on gut health and inflammation, their overconsumption could provoke adverse neurological outcomes, particularly in susceptible populations.

Moreover, SCFAs can influence immune responses in ways that may not always be beneficial. For example, while butyrate is known to promote regulatory T cell (Treg) differentiation, it can also enhance the expression of pro-inflammatory cytokines in certain contexts (Nomura et al., 2020; Arpaia et al., 2013). This duality indicates that SCFA treatment might inadvertently lead to an imbalance in immune homeostasis. Therefore, potentially aggravating autoimmune conditions or inflammatory diseases. In cancer therapy, high levels of butyrate have been shown to limit the efficacy of immune checkpoint inhibitors, suggesting that SCFAs might dampen anti-tumor immune responses (Coutzac et al., 2020; Malczewski et al., 2021). However, it has been reported that a minimal inflammatory state is essential for maintaining microbiota homeostasis (Brooks et al., 2021). The circadian-dependent regulation of innate immune processes has been shown to be effective and plays a protective role under normal physiological conditions. This circadian regulation ensures that the immune system is appropriately activated at specific times, thereby supporting the body’s ability to respond to external threats while preventing excessive inflammation that could lead to tissue damage.

Irregular SCFA levels could lead to further complications in metabolic health (Segers et al., 2018; Fawad et al., 2022). For instance, studies have shown that circadian disruption can exacerbate inflammatory bowel disease (IBD) symptoms, suggesting that the timing and dosage of SCFA treatment must be carefully considered in individuals with existing health issues (Jochum et al., 2022). Furthermore, high concentrations of SCFAs can lead to gastrointestinal discomfort, including diarrhea and bloating, particularly if the gut microbiota is not well-adapted to increased SCFA levels (De La Cuesta-Zuluaga et al., 2018). This discomfort may discourage adherence to dietary interventions aimed at improving metabolic health, ultimately undermining the benefits of SCFA supplementation. Additionally, dysbiosis resulting from excessive SCFA intake can compromise the intestinal barrier, increasing the risk of systemic inflammation and related health issues (Jochum et al., 2022).

In an article about chronotype across night workers, evidence supported that the individual chronotype is linked to reduced health damage caused by night shifts (Akimova et al., 2023). The polygenic score for eveningness was used to measure chronotype. The results of this study suggest that people who are genetically predisposed to going to bed later than others have a lower risk of health damage from night shift work. Genetic polymorphisms in *CLOCK* and *PER3* have since been implicated in psychiatric disorders such as bipolar disorder, major depressive disorder, and attention-deficit hyperactivity disorder (ADHD) (Janoski et al., 2024). Individual differences in these genetic variants may affect circadian regulation, which when dysregulated can aggravate mood and cognitive manifestations (Ferrer et al., 2020; Shi et al., 2016; Schuch et al., 2018). These findings call for the consideration of circadian biology in psychiatric pathogenesis, as well as the practical control of temporal niches for psychiatric disorders especially for night shift workers who undergo circadian disruption.

### 4.4 Future directions in SCFA research and clinical translation

The current gaps in knowledge need to be addressed, particularly the lack of low-dose studies in humans, as animal models do not fully recapitulate the physiological responses seen in humans. Thus, adequate quality clinical studies in diverse cohorts are needed to confirm findings based on animal models. Furthermore, clinical studies would be necessary to confirm the therapeutic reliability of SCFA supplementation and its ability to modulate peripheral circadian rhythms in the clinical environment.

SCFA administration should also be further studied for its long-term metabolic and hormonal effects. It will be important to characterize the safety profile of SCFA supplementation with a focus on evaluating the risk and frequency of adverse events and undesirable consequences. Furthermore, investigating the potential for SCFA supplementation to act in synergy with modifiable lifestyle factors (for example, regular sleep/wake cycles and dietary modulation) could be employed to establish a more individualized circadian rhythm modulation strategy and an integral therapeutic approach. Deciphering the molecular mechanisms mediating the gut microbiota-peripheral circadian clock axis is critical for informing targeted, personalized therapeutic approaches. Such developments could help those with circadian misalignment due to these causes, as well as other risks such as metabolic disorders, and create new opportunities for precision medicine strategies.

### 4.5 Limitations

Our study has several limitations. First, this systematic review includes animal studies, which presents challenges in data synthesis and methodological uniformity across species. Moreover, valuable insights from animal studies are not necessarily applicable to humans. However, due to the lack of human studies in this field, our search was extended to animal and *in vitro* studies. Second, the risk of bias assessment in animal studies showed many instances of an unclear risk of bias across various domains, particularly regarding blinding and randomization, as often happens in animal experimental studies. Another limitation was the lack of studies on interventions with SCFA supplementation. Fluctuations in SCFA levels can be associated with various causes; therefore, it is more substantial to use oral or intraperitoneal administration to manually increase SCFA concentration. Addressing these limitations in future research is crucial for further developments and potentially impactful clinical applications.

## 5 Conclusion

To our knowledge, this review is the first to point out the possibility of SCFAs modulating peripheral clock gene expression, such as *PER1*, *PER2*, *CRY1*, *CRY2*, and *BMAL1*. The results indicate that SCFA levels are strongly related to peripheral circadian gene expression changes, bringing new insights into their possible clinical use in circadian rhythm regulation. However, because most of the studies discussed here were conducted in animal models, more well-designed human studies with diverse populations are needed to validate the existing findings. Risk of bias assessment revealed several domains with unclear risk, pointing out the need for stricter methodologies for future studies.

SCFA supplementation is promising, although its long-term impact needs to be more closely examined. Although the current evidence does not demonstrate significant risks, the safety profile of SCFA supplementation is based on limited data, necessitating further investigation. SCFA supplementation is not likely to be a monotherapy but part of a multimodal health intervention integrating diet, exercise, and other lifestyle interventions. This is particularly applicable to individuals with obesity, type 2 diabetes, or cardiovascular disorders in whom circadian misalignment can contribute to worsening disease and symptoms. Such techniques must be used to augment pharmacological interventions where required, with the aim of providing a holistic strategy to the treatment of circadian rhythm disorders. Future studies also need to prioritize the optimization of supplementation regimens and gene expression analysis tools to improve study quality. Further, examining SCFA interactions within the gut microbiota-circadian axis may yield important information regarding its therapeutic potential more generally.

## Data Availability

The original contributions presented in the study are included in the article/Supplementary Material, further inquiries can be directed to the corresponding author.
