# Supplementary material for: The modulatory role of short-chain fatty acids on peripheral circadian gene expression: a systematic review
Source: Front Physiol. 2025 Jul 14;16:1595057. doi: 10.3389/fphys.2025.1595057 (PMC12301408; doi:10.3389/fphys.2025.1595057)
Supplement: Supplementary file 2 [file Table3.docx]

# SYRCLE Risk of Bias

| **Authors** | **D1** | **D2** | **D3** | **D4** | **D5** | **D6** | **D7** | **D8** | **D9** | **D10** |
| --- | --- | --- | --- | --- | --- | --- | --- | --- | --- | --- |
| Tahara et al. | U | L | U | L | U | U | U | L | L | L |
| Ding et al. | L | L | L | L | U | U | H | L | U | L |
| Leone et al. | U | L | L | L | U | U | H | L | U | L |
| Ryan et al. | L | L | U | L | U | U | U | L | L | L |
| Altaha et al. | U | U | L | L | U | U | H | L | U | U |
| Fawad et al. | U | U | L | L | U | U | H | L | U | U |
| Desmet et al. | L | L | L | L | U | L | U | L | L | L |

Judgment:

L: Low Risk of Bias

U: Unclear Risk of Bias

H: High Risk of Bias

Domains:

- D1: Sequence generation
- D2: Baseline characteristics
- D3: Allocation concealment
- D4: Random housing
- D5: Blinding of caregivers
- D6: Random outcome assessment
- D7: Blinding of outcome assessor
- D8: Incomplete outcome data
- D9: Selective outcome reporting
- D10: Other sources of bias
