# Supplementary material for: The modulatory role of short-chain fatty acids on peripheral circadian gene expression: a systematic review
Source: Front Physiol. 2025 Jul 14;16:1595057. doi: 10.3389/fphys.2025.1595057 (PMC12301408; doi:10.3389/fphys.2025.1595057)
Supplement: Supplementary file 4 [file Table4.docx]

# RoB 2 Risk of bias

| **Study** | **D1** | **D2** | **D3** | **D4** | **D5** | **Overall** |
| --- | --- | --- | --- | --- | --- | --- |
| Firoozi et al. | **L** | **L** | **U** | **L** | **L** | **L** |

Domains:
D1: Bias arising from the randomization process.
D2: Bias due to deviations from intended intervention.
D3: Bias due to missing outcome data.
D4: Bias in measurement of the outcome.
D5: Bias in selection of the reported result.

Judgment:
H - High Risk, U - Unclear Risk, L - Low Risk
