# Supplementary material for: The modulatory role of short-chain fatty acids on peripheral circadian gene expression: a systematic review
Source: Front Physiol. 2025 Jul 14;16:1595057. doi: 10.3389/fphys.2025.1595057 (PMC12301408; doi:10.3389/fphys.2025.1595057)
Supplement: Supplementary file 5 [file DataSheet1.docx]

Supplementary Material

# Supplementary Data Scite.ai input prompts and outcome

# Prompt:

I wrote a literature review about how SCFA effects peripheral circadian gene expression. Write to me about the potential risk of SCFA treatment.

**Outcome:**

Short-chain fatty acids (SCFAs) have garnered attention for their potential therapeutic benefits, particularly in modulating immune responses and influencing metabolic pathways. However, treatment with SCFAs is not without risks, which can manifest in various physiological and pathological contexts.

One significant concern is the potential for SCFA treatment to exacerbate certain neurological conditions. For instance, excessive propionate intake has been linked to an increased risk of Alzheimer's disease (AD) in healthy individuals, as it may lead to microglial activation and neuroinflammation (Xie et al., 2023; Killingsworth et al., 2021). This suggests that while SCFAs can have beneficial effects on gut health and inflammation, their overconsumption could provoke adverse neurological outcomes, particularly in susceptible populations.

Moreover, SCFAs can influence immune responses in ways that may not always be beneficial. For example, while butyrate is known to promote regulatory T cell (Treg) differentiation, it can also enhance the expression of pro-inflammatory cytokines in certain contexts (Nomura et al., 2020; Arpaia et al., 2013). This duality indicates that SCFA treatment might inadvertently lead to an imbalance in immune homeostasis, potentially aggravating autoimmune conditions or inflammatory diseases. In cancer therapy, high levels of butyrate have been shown to limit the efficacy of immune checkpoint inhibitors, suggesting that SCFAs might dampen anti-tumor immune responses (Coutzac et al., 2020; Malczewski et al., 2021).

Furthermore, the systemic effects of SCFAs, particularly their ability to cross the blood-brain barrier, raise concerns about unintended consequences on brain function and behavior. Although SCFAs can exert neuroprotective effects, their dysregulated levels may influence neuroinflammatory processes, potentially exacerbating conditions such as autism spectrum disorders or other neurodevelopmental issues (Koh et al., 2016; MacFabe, 2015).

In conclusion, while SCFAs present promising therapeutic avenues, their treatment must be approached with caution. The potential risks associated with SCFA treatment, including exacerbation of neurological conditions, immune dysregulation, dysbiosis, and unintended systemic effects, necessitate further research to delineate safe and effective therapeutic protocols.

**Prompt:**

Dive deeper in doses and more into a particular sample of people with circadian rhythms misalignment, such as night shift workers.

**Outcome:**

The treatment of circadian rhythm misalignment, particularly in populations such as night shift workers, presents unique challenges and potential risks. Night shift work is associated with significant alterations in metabolic processes, including glucose metabolism, which can be exacerbated by the consumption of short-chain fatty acids (SCFAs) as a therapeutic intervention.

Research indicates that night shift workers often experience internal circadian misalignment, leading to impaired glucose tolerance and insulin sensitivity (Chellappa et al., 2021; Morris et al., 2015). This misalignment is particularly pronounced in individuals with early chronotypes, who may struggle more with night shifts due to their natural sleep-wake preferences (Marqueze et al., 2021). The timing of food intake is crucial; studies have shown that rescheduling meals to align with night work can prevent some metabolic disturbances, yet this practice can also lead to increased risks of metabolic syndrome and type 2 diabetes due to the inherent misalignment of circadian rhythms (Chellappa et al., 2021; Morris et al., 2015).

SCFAs, produced by gut microbiota, have been shown to influence circadian rhythms and may offer therapeutic benefits in this context. For instance, SCFAs can induce circadian clock entrainment in peripheral tissues, which could theoretically help mitigate some of the adverse effects of circadian misalignment (Tahara et al., 2018; Han et al., 2021). However, the chronic administration of SCFAs in night shift workers is not without risks. Excessive SCFA levels can lead to gastrointestinal discomfort and dysbiosis, potentially worsening the metabolic disturbances associated with shift work (Tahara et al., 2018; Khan et al., 2020). Furthermore, while SCFAs can promote beneficial effects on gut health and circadian regulation, their impact on systemic inflammation and immune responses remains a concern, particularly in populations already at risk due to their work schedules (Jochum et al., 2022).

Moreover, the interplay between SCFAs and the circadian system is complex. SCFAs have been implicated in the modulation of circadian gene expression, which can be beneficial; however, disruptions in this modulation due to irregular SCFA levels could lead to further complications in metabolic health (Segers et al., 2018; Luzader et al., 2020). For instance, studies have shown that circadian disruption can exacerbate inflammatory bowel disease (IBD) symptoms, suggesting that the timing and dosage of SCFA treatment must be carefully considered in individuals with existing health issues (Jochum et al., 2022).

In conclusion, while SCFAs may hold promise for addressing some of the metabolic challenges faced by night shift workers, their administration must be approached with caution. The potential for exacerbating metabolic disorders, gastrointestinal issues, and inflammatory responses necessitates a thorough understanding of individual health profiles and careful monitoring of SCFA levels. Future research should focus on optimizing SCFA treatment protocols to balance their benefits against the risks associated with circadian misalignment.

**Prompt:**

It is known that SCFA can influence circadian rhythms with G-protein-coupled receptor (GPCR) activation, epigenetic regulation via histone deacetylase (HDAC) inhibition, and metabolic hormone signaling. Describe the potential risk that can occur after SCfA supplementation targets those pathways.

**Outcome:**

Short-chain fatty acids (SCFAs) have been recognized for their multifaceted roles in influencing circadian rhythms through various mechanisms, including G-protein-coupled receptor (GPCR) activation, epigenetic regulation via histone deacetylase (HDAC) inhibition, and modulation of metabolic hormone signaling. However, the supplementation of SCFAs can pose potential risks, particularly when targeting these pathways.

### GPCR Activation and Metabolic Hormone Signaling

SCFAs, such as butyrate and propionate, exert their effects primarily through the activation of GPCRs, notably FFAR2 and FFAR3, which are involved in the regulation of metabolic hormones like glucagon-like peptide-1 (GLP-1) (Tolhurst et al., 2012; Kasubuchi et al., 2015). While this activation can enhance insulin sensitivity and promote satiety, excessive SCFA levels may lead to dysregulation of these hormones. For example, overactivation of FFAR2 can result in impaired glucose homeostasis, potentially contributing to insulin resistance and metabolic syndrome (Tolhurst et al., 2012; Kopczyńska & Kowalczyk, 2024). Furthermore, the chronic stimulation of these receptors may lead to desensitization, diminishing their effectiveness over time and possibly exacerbating metabolic disorders (Kasubuchi et al., 2015).

### Epigenetic Regulation via HDAC Inhibition

SCFAs are potent inhibitors of HDACs, leading to increased acetylation of histones and subsequent alterations in gene expression. This mechanism is crucial for the differentiation of regulatory T cells and the modulation of inflammatory responses (Tahara et al., 2018). However, excessive HDAC inhibition can disrupt normal cellular functions and lead to aberrant gene expression patterns. For instance, prolonged SCFA supplementation may result in the upregulation of pro-inflammatory genes, which could counteract the intended anti-inflammatory effects of SCFAs (Luzader et al., 2020). Additionally, the dysregulation of circadian genes due to altered histone acetylation could lead to further disruptions in circadian rhythms, compounding the risks associated with SCFA supplementation (Tahara et al., 2018; Jochum et al., 2022).

### Circadian Rhythm Disruption

The administration of SCFAs can also influence circadian rhythms directly. Research indicates that SCFAs can modulate the expression of circadian clock genes, which are critical for maintaining metabolic homeostasis (Gao et al., 2020; Segers et al., 2018). However, if SCFA supplementation is misaligned with the body's natural circadian rhythms—such as in night shift workers or individuals with irregular eating patterns—this could exacerbate circadian misalignment and lead to metabolic dysregulation (Desmet et al., 2021; Ding et al., 2022). For example, the timing of SCFA intake may disrupt the diurnal rhythms of gut microbiota and their metabolites, leading to further complications in metabolic health (Zhen et al., 2022).

### Gastrointestinal and Systemic Effects

Moreover, the gastrointestinal effects of SCFA supplementation cannot be overlooked. High concentrations of SCFAs can lead to gastrointestinal discomfort, including diarrhea and bloating, particularly if the gut microbiota is not well-adapted to increased SCFA levels (Cuesta‐Zuluaga et al., 2018). This discomfort may discourage adherence to dietary interventions aimed at improving metabolic health, ultimately undermining the benefits of SCFA supplementation. Additionally, dysbiosis resulting from excessive SCFA intake can compromise the intestinal barrier, increasing the risk of systemic inflammation and related health issues (Jochum et al., 2022).

### Conclusion

In summary, while SCFAs hold promise for modulating circadian rhythms and improving metabolic health, their supplementation must be approached with caution. The potential risks associated with GPCR activation, HDAC inhibition, circadian disruption, and gastrointestinal effects necessitate a careful consideration of dosage, timing, and individual health status. Future research should aim to elucidate the optimal conditions for SCFA supplementation to maximize benefits while minimizing risks.

**Prompt:**

Describe to me in details molecular Mechanisms of SCFA modulation effect on peripheral circadian genes through gut microbiota, GPR41/GPR43 receptors, HDAC inhibition, TFG-β signaling through pH fluctuation, and hormone secretion (GLP-1, insulin)

**Outcome:**

Short-chain fatty acids (SCFAs), primarily produced by gut microbiota during the fermentation of dietary fibers, have emerged as significant modulators of various physiological processes, including the regulation of peripheral circadian genes. The molecular mechanisms underlying these effects involve several pathways, including the activation of GPR41/GPR43 receptors, inhibition of histone deacetylases (HDACs), modulation of TGF-β signaling, and the regulation of hormone secretion such as GLP-1 and insulin.

**GPR41/GPR43 Receptors and SCFA Signaling**

SCFAs such as acetate, propionate, and butyrate exert their effects primarily through G protein-coupled receptors (GPCRs) GPR41 and GPR43. These receptors are expressed in various tissues, including the gut and immune cells, and their activation leads to a cascade of intracellular signaling events that influence gene expression related to metabolism and circadian rhythms. For instance, the activation of GPR41 has been shown to enhance the differentiation and function of regulatory T cells, which play a crucial role in maintaining immune homeostasis and influencing metabolic processes (Hu et al., 2022). Furthermore, SCFAs can modulate the expression of circadian genes, thereby impacting the timing of metabolic processes (Nagata et al., 2024).

**HDAC Inhibition and Epigenetic Modulation**

One of the critical mechanisms by which SCFAs influence gene expression is through the inhibition of HDACs. SCFAs, particularly butyrate, have been demonstrated to inhibit HDAC activity, leading to an increase in histone acetylation and subsequent transcriptional activation of target genes (Folkerts et al., 2020). This epigenetic modification is particularly relevant in immune cells, where SCFAs can enhance the expression of anti-inflammatory cytokines and regulatory genes, thereby modulating immune responses and potentially influencing circadian rhythms (Tang et al., 2010). The interplay between SCFAs and HDACs highlights the importance of epigenetic regulation in the context of gut microbiota and circadian biology.

**TGF-β Signaling Pathway**

The TGF-β signaling pathway is another critical mediator of the effects of SCFAs on circadian gene expression. SCFAs can enhance TGF-β signaling through various mechanisms, including the modulation of receptor availability and intracellular signaling cascades. For instance, insulin signaling has been shown to enhance the delivery of TGF-β receptors to the cell surface, thereby increasing TGF-β responsiveness (Budi et al., 2015). This integration of insulin and TGF-β signaling pathways suggests a complex regulatory network where SCFAs may influence metabolic responses through TGF-β-mediated pathways. Additionally, TGF-β signaling is known to regulate various cellular processes, including cell growth, differentiation, and apoptosis, which are essential for maintaining circadian rhythms (Ikushima & Miyazono, 2011).

**Hormonal Regulation: GLP-1 and Insulin**

SCFAs also play a significant role in the secretion of hormones such as GLP-1 and insulin, which are crucial for glucose metabolism and circadian regulation. The secretion of GLP-1 from intestinal L-cells is stimulated by SCFAs, particularly butyrate and propionate, leading to enhanced insulin secretion from pancreatic β-cells (Nagata et al., 2024; Budi et al., 2019). This hormonal interplay is vital for maintaining glucose homeostasis and can influence circadian rhythms by modulating energy metabolism and feeding behavior. Furthermore, the interaction between SCFAs and insulin signaling pathways underscores the importance of gut microbiota in regulating metabolic health and circadian biology (Budi et al., 2019).

In summary, SCFAs exert their modulatory effects on peripheral circadian genes through a multifaceted approach involving GPR41/GPR43 receptor activation, HDAC inhibition, TGF-β signaling modulation, and the regulation of hormone secretion. These mechanisms illustrate the intricate relationship between gut microbiota, metabolic regulation, and circadian biology, highlighting the potential for therapeutic interventions targeting SCFA pathways in metabolic disorders.

**Prompt:**

Dive deeper into molecular aspect

**Outcome:**

The modulation of peripheral circadian genes by short-chain fatty acids (SCFAs) through gut microbiota involves intricate molecular mechanisms, including the activation of GPR41/GPR43 receptors, inhibition of histone deacetylases (HDACs), modulation of TGF-β signaling, and the regulation of hormone secretion such as GLP-1 and insulin. Each of these pathways contributes to the overall impact of SCFAs on metabolic processes and circadian rhythms.

**GPR41/GPR43 Receptors and SCFA Signaling**

SCFAs, particularly acetate, propionate, and butyrate, interact with G protein-coupled receptors (GPCRs) such as GPR41 and GPR43. These receptors are predominantly expressed in intestinal L-cells, where they play a crucial role in stimulating the secretion of GLP-1, a hormone that enhances insulin secretion and regulates glucose homeostasis (Kimura et al., 2013; Tolhurst et al., 2012). GPR43 has been shown to mediate GLP-1 secretion via Gq protein signaling, while GPR41 primarily signals through Gi/o proteins (Kimura et al., 2013). This receptor-mediated pathway is essential for the physiological effects of SCFAs, including their influence on energy metabolism and circadian rhythm regulation (Tolhurst et al., 2012).

Moreover, the activation of GPR43 in adipose tissue has been linked to the modulation of fat accumulation and insulin sensitivity, highlighting the systemic effects of SCFAs beyond the gut (Kimura et al., 2013). The interplay between SCFAs and these receptors underscores the importance of gut microbiota in maintaining metabolic health and circadian synchronization.

**HDAC Inhibition and Epigenetic Modulation**

SCFAs, particularly butyrate, are known to inhibit HDACs, leading to increased histone acetylation and subsequent transcriptional activation of target genes (Folkerts et al., 2020; Tang et al., 2010). This epigenetic modification is crucial for the regulation of genes involved in inflammation and metabolism, which are often linked to circadian rhythms. For instance, the inhibition of HDACs by SCFAs can enhance the expression of circadian genes, thereby influencing metabolic processes that follow a daily rhythm (Folkerts et al., 2020). The role of HDAC inhibition in modulating immune responses and metabolic pathways further illustrates the significance of SCFAs in circadian biology (Tang et al., 2010).

**TGF-β Signaling Pathway**

The TGF-β signaling pathway is another critical mechanism through which SCFAs exert their effects. SCFAs can enhance TGF-β signaling, which is essential for various cellular processes, including growth, differentiation, and apoptosis (Budi et al., 2015; Ikushima & Miyazono, 2011). This pathway is particularly relevant in the context of insulin signaling, as TGF-β can modulate the responsiveness of cells to insulin, thereby influencing glucose metabolism and circadian regulation (Budi et al., 2015; Ikushima & Miyazono, 2011). The integration of TGF-β signaling with insulin pathways suggests a complex regulatory network where SCFAs can influence both metabolic and circadian rhythms (Budi et al., 2019).

**Hormonal Regulation: GLP-1 and Insulin**

SCFAs significantly influence the secretion of hormones such as GLP-1 and insulin, which are crucial for glucose homeostasis and metabolic regulation. The secretion of GLP-1 from intestinal L-cells is stimulated by SCFAs through the activation of GPR41 and GPR43 (Tolhurst et al., 2012; Psichas et al., 2014). This hormonal response is vital for enhancing insulin secretion from pancreatic β-cells, thereby contributing to the incretin effect that helps regulate blood glucose levels postprandially (Kume et al., 2018; Cho et al., 2014). Additionally, SCFAs have been shown to increase the number of L-cells in the intestinal epithelium, further amplifying GLP-1 secretion and its metabolic effects (Rahat-Rozenbloom et al., 2016).

Furthermore, the interplay between SCFAs and insulin signaling pathways underscores the importance of gut microbiota in regulating metabolic health and circadian biology. The secretion of insulin, influenced by GLP-1 and SCFAs, plays a crucial role in maintaining energy balance and synchronizing metabolic processes with circadian rhythms (Cho et al., 2014; Kim & Schuppan, 2012).

**Conclusion**

In conclusion, the molecular mechanisms by which SCFAs modulate peripheral circadian genes are multifaceted and involve the activation of GPR41/GPR43 receptors, inhibition of HDACs, modulation of TGF-β signaling, and regulation of hormone secretion such as GLP-1 and insulin. These pathways illustrate the intricate relationship between gut microbiota, metabolic regulation, and circadian biology, highlighting the potential for therapeutic interventions targeting SCFA pathways in metabolic disorders.

**Prompt:**

Focus only on GPR41/GPR43 Receptors and SCFA Signaling

**Outcome:**

The role of short-chain fatty acids (SCFAs) in modulating peripheral circadian genes through the activation of GPR41 and GPR43 receptors is a critical area of research, particularly in understanding how gut microbiota influences metabolic processes and circadian rhythms. SCFAs, primarily produced by the fermentation of dietary fibers by gut microbiota, activate these G protein-coupled receptors (GPCRs), leading to various physiological effects.

**GPR41 and GPR43 Receptors: Structure and Function**

GPR41 (also known as FFAR3) and GPR43 (FFAR2) are GPCRs that respond to SCFAs such as acetate, propionate, and butyrate. These receptors are expressed in various tissues, including the gastrointestinal tract, adipose tissue, and immune cells, indicating their widespread influence on metabolic regulation (Kim et al., 2014; Kimura et al., 2013). The activation of GPR41 and GPR43 by SCFAs initiates intracellular signaling cascades that can modulate gene expression, particularly those involved in energy metabolism and inflammation (Eberle et al., 2014; Kimura et al., 2013).

GPR41 has a higher affinity for propionate and butyrate compared to acetate, while GPR43 exhibits comparable agonist activities for all three SCFAs (Li et al., 2012; Mikami et al., 2020). This differential specificity suggests that the physiological effects of SCFAs may vary depending on the receptor activated and the tissue context. For instance, GPR43 activation has been linked to the modulation of appetite and energy expenditure, while GPR41 is involved in sympathetic nervous system activation, influencing energy homeostasis (Kimura et al., 2013; Kimura et al., 2011).

**Intracellular Signaling Pathways**

Upon activation by SCFAs, GPR41 and GPR43 engage distinct intracellular signaling pathways. GPR43 primarily signals through the Gαi and Gαq families of heterotrimeric G proteins, leading to a decrease in cyclic AMP (cAMP) levels and an increase in intracellular calcium concentrations (Park et al., 2021; Mikami et al., 2020). This signaling can result in various cellular responses, including the release of hormones such as GLP-1 and PYY, which are crucial for regulating appetite and glucose metabolism (Yang et al., 2022; Cornall et al., 2011).

Moreover, the activation of GPR41 has been shown to influence the sympathetic nervous system, promoting energy expenditure and fat oxidation (Kimura et al., 2013; Kimura et al., 2011). This suggests that SCFAs not only serve as energy sources but also act as signaling molecules that communicate metabolic status to various tissues through these receptors.

**Physiological Implications of GPR41/GPR43 Activation**

The physiological implications of SCFA signaling through GPR41 and GPR43 are profound. For instance, the activation of these receptors has been associated with anti-inflammatory effects, as they can modulate immune cell function and cytokine production (Dinakis et al., 2021; Imoto et al., 2018). In particular, GPR43 activation has been linked to the polarization of T lymphocytes towards a regulatory phenotype, which is essential for maintaining immune homeostasis (Wang et al., 2019; Dinakis et al., 2021).

Furthermore, the role of SCFAs in regulating lipid metabolism is significant. Studies have shown that SCFA signaling through GPR41 and GPR43 can inhibit fat accumulation and promote lipid oxidation, thereby influencing body weight and metabolic health (Kim et al., 2014; Kimura et al., 2013). This is particularly relevant in the context of obesity and metabolic syndrome, where dysregulation of SCFA production and receptor signaling may contribute to the pathophysiology of these conditions.

**Conclusion**

In summary, the activation of GPR41 and GPR43 receptors by SCFAs plays a crucial role in modulating peripheral circadian genes and influencing various metabolic processes. The differential specificity of these receptors for SCFAs, coupled with their ability to engage distinct intracellular signaling pathways, underscores their importance in the communication between gut microbiota and host metabolism. Understanding these mechanisms may provide insights into potential therapeutic strategies for metabolic disorders and circadian rhythm disruptions.

**Prompt:**

Quantitative real-time PCR (qRT-PCR), Bioluminescence imaging in PER2::LUC reporter mice, 16S rRNA amplicon sequencing analysis. Compare these methodologies for measuring circadian gene expression.

**Outcome:**

Quantitative real-time PCR (qRT-PCR), bioluminescence imaging in PER2::LUC reporter mice, and 16S rRNA amplicon sequencing are three distinct methodologies employed to measure circadian gene expression, each with its unique advantages and limitations.

qRT-PCR is a widely utilized technique for quantifying gene expression levels, including circadian genes. It allows for the precise measurement of mRNA levels, providing insights into the temporal dynamics of gene expression. This method is highly sensitive and can detect low abundance transcripts, making it suitable for analyzing circadian rhythms at specific time points (Koo et al., 2015). However, qRT-PCR requires prior knowledge of the gene of interest and is limited to a predefined set of genes, which may not capture the full complexity of circadian regulation across the genome (Yoo et al., 2017). Additionally, the technique does not provide information on the spatial distribution of gene expression within tissues (Martin-Burgos et al., 2021).

In contrast, bioluminescence imaging using PER2::LUC reporter mice offers a real-time, in vivo assessment of circadian gene expression. This method exploits the bioluminescent properties of luciferase, allowing researchers to monitor the oscillation of PER2 expression in living organisms. Bioluminescence imaging can reveal the dynamics of circadian rhythms across different tissues and provide insights into the anatomical sources of circadian signals (Ding et al., 2022). For instance, studies have shown that bioluminescence signals can originate from various organs, including the kidneys and liver, which are crucial for understanding the systemic nature of circadian rhythms (Taylor et al., 2019). However, this method may not provide the same level of specificity as qRT-PCR, as it measures the overall bioluminescence rather than specific mRNA levels (Baba et al., 2017).

16S rRNA amplicon sequencing is primarily used to analyze microbial communities and their interactions with host circadian rhythms. This technique allows for the identification and characterization of gut microbiota, which has been shown to influence circadian rhythms through various mechanisms, including the production of metabolites that can affect host gene expression (Shin et al., 2016). The 16S rRNA sequencing approach provides a comprehensive view of microbial diversity and can reveal temporal changes in microbial communities in relation to circadian cycles (Milićević et al., 2024). However, it is important to note that while 16S rRNA sequencing can indicate community composition, it may lack the resolution to differentiate closely related species due to the conserved nature of the 16S rRNA gene (Ono et al., 2015). Furthermore, the methodology is primarily focused on microbial analysis and does not directly measure circadian gene expression in host tissues (Taylor et al., 2020).

In summary, while qRT-PCR provides high sensitivity and specificity for measuring circadian gene expression at the mRNA level, bioluminescence imaging offers a dynamic view of gene expression in vivo across various tissues. On the other hand, 16S rRNA amplicon sequencing provides insights into the microbial influence on circadian rhythms but does not directly measure host gene expression. Each methodology has its strengths and limitations, and their combined use could provide a more comprehensive understanding of circadian gene regulation and its implications for health and disease.

# Supplementary Figures and Tables Supplementary Table 1


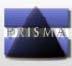
 **PRISMA 2020 Checklist**

| **Section and Topic** | **Item #** | **Checklist item** | **Location where item is reported**  **(Page)** |
| --- | --- | --- | --- |
| **TITLE** | | |  |
| Title | 1 | Identify the report as a systematic review. | 1 |
| **ABSTRACT** | | |  |
| Abstract | 2 | See the PRISMA 2020 for Abstracts checklist. | 1 |
| **INTRODUCTION** | | |  |
| Rationale | 3 | Describe the rationale for the review in the context of existing knowledge. | 1-5 |
| Objectives | 4 | Provide an explicit statement of the objective(s) or question(s) the review addresses. | 3 and 6 |
| **METHODS** | | |  |
| Eligibility criteria | 5 | Specify the inclusion and exclusion criteria for the review and how studies were grouped for the syntheses. | 6-7 |
| Information sources | 6 | Specify all databases, registers, websites, organisations, reference lists and other sources searched or consulted to identify studies. Specify the date when each source was last searched or consulted. | 6-7 |
| Search strategy | 7 | Present the full search strategies for all databases, registers and websites, including any filters and limits used. | 6-7 |
| Selection process | 8 | Specify the methods used to decide whether a study met the inclusion criteria of the review, including how many reviewers screened each record and each report retrieved, whether they worked independently, and if applicable, details of automation tools used in the process. | 6-7 |
| Data collection process | 9 | Specify the methods used to collect data from reports, including how many reviewers collected data from each report, whether they worked independently, any processes for obtaining or confirming data from study investigators, and if applicable, details of automation tools used in the process. | 6-7 |
| Data items | 10a | List and define all outcomes for which data were sought. Specify whether all results that were compatible with each outcome domain in each study were sought (e.g. for all measures, time points, analyses), and if not, the methods used to decide which results to collect. | 6-7 |
|  | 10b | List and define all other variables for which data were sought (e.g. participant and intervention characteristics, funding sources). Describe any assumptions made about any missing or unclear information. | 6-7 |
| Study risk of bias assessment | 11 | Specify the methods used to assess risk of bias in the included studies, including details of the tool(s) used, how many reviewers assessed each study and whether they worked independently, and if applicable, details of automation tools used in the process. | 6-7 |
| Effect measures | 12 | Specify for each outcome the effect measure(s) (e.g. risk ratio, mean difference) used in the synthesis or presentation of results. | 6-7 |
| Synthesis methods | 13a | Describe the processes used to decide which studies were eligible for each synthesis (e.g. tabulating the study intervention characteristics and comparing against the planned groups for each synthesis (item #5)). | 6-7 |
|  | 13b | Describe any methods required to prepare the data for presentation or synthesis, such as handling of missing summary statistics, or data conversions. | 6-7 |
|  | 13c | Describe any methods used to tabulate or visually display results of individual studies and syntheses. | 6-7 |
|  | 13d | Describe any methods used to synthesize results and provide a rationale for the choice(s). If meta-analysis was performed, describe the model(s), method(s) to identify the presence and extent of statistical heterogeneity, and software package(s) used. | 6-7 |
|  | 13e | Describe any methods used to explore possible causes of heterogeneity among study results (e.g. subgroup analysis, meta-regression). | 6-7 |
|  | 13f | Describe any sensitivity analyses conducted to assess robustness of the synthesized results. | 6-7 |
| Reporting bias assessment | 14 | Describe any methods used to assess risk of bias due to missing results in a synthesis (arising from reporting biases). | 6-7 |
| Certainty assessment | 15 | Describe any methods used to assess certainty (or confidence) in the body of evidence for an outcome. | N/A |
| **RESULTS** | | |  |
| Study selection | 16a | Describe the results of the search and selection process, from the number of records identified in the search to the number of studies included in the review, ideally using a flow diagram. | 7 |
|  | 16b | Cite studies that might appear to meet the inclusion criteria, but which were excluded, and explain why they were excluded. | 7 |
| Study characteristics | 17 | Cite each included study and present its characteristics. | 7-10 |
| Risk of bias in studies | 18 | Present assessments of risk of bias for each included study. | 10-11 |
| Results of individual studies | 19 | For all outcomes, present, for each study: (a) summary statistics for each group (where appropriate) and (b) an effect estimate and its precision (e.g. confidence/credible interval), ideally using structured tables or plots. | 7-11 |
| Results of syntheses | 20a | For each synthesis, briefly summarise the characteristics and risk of bias among contributing studies. | 7-11 |
|  | 20b | Present results of all statistical syntheses conducted. If meta-analysis was done, present for each the summary estimate and its precision (e.g. confidence/credible interval) and measures of statistical heterogeneity. If comparing groups, describe the direction of the effect. | 7-11 |
|  | 20c | Present results of all investigations of possible causes of heterogeneity among study results. | 7-11 |
|  | 20d | Present results of all sensitivity analyses conducted to assess the robustness of the synthesized results. | N/A |
| Reporting biases | 21 | Present assessments of risk of bias due to missing results (arising from reporting biases) for each synthesis assessed. | N/A |
| Certainty of evidence | 22 | Present assessments of certainty (or confidence) in the body of evidence for each outcome assessed. | N/A |
| **DISCUSSION** | | |  |
| Discussion | 23a | Provide a general interpretation of the results in the context of other evidence. | 11-14 |
|  | 23b | Discuss any limitations of the evidence included in the review. | 11-14 |
|  | 23c | Discuss any limitations of the review processes used. | 15 |
|  | 23d | Discuss implications of the results for practice, policy, and future research. | 15 |
| **OTHER INFORMATION** | | |  |
| Registration and protocol | 24a | Provide registration information for the review, including register name and registration number, or state that the review was not registered. | 6 |
|  | 24b | Indicate where the review protocol can be accessed, or state that a protocol was not prepared. | 6 |
|  | 24c | Describe and explain any amendments to information provided at registration or in the protocol. | 6 |
| Support | 25 | Describe sources of financial or non-financial support for the review, and the role of the funders or sponsors in the review. | 16 |
| Competing interests | 26 | Declare any competing interests of review authors. | 16 |
| Availability of data, code and other materials | 27 | Report which of the following are publicly available and where they can be found: template data collection forms; data extracted from included studies; data used for all analyses; analytic code; any other materials used in the review. | 16-26 |

*From:*  Page MJ, McKenzie JE, Bossuyt PM, Boutron I, Hoffmann TC, Mulrow CD, et al. The PRISMA 2020 statement: an updated guideline for reporting systematic reviews. BMJ 2021;372:n71. doi: 10.1136/bmj.n71. This work is licensed under CC BY 4.0. To view a copy of this license, visit <https://creativecommons.org/licenses/by/4.0/>
